# Corrosion Inhibition of API 5L X60 Steel in Acid Medium: Theoretical and Experimental Approaches

**DOI:** 10.1021/acsomega.5c00751

**Published:** 2025-04-28

**Authors:** Paulina Arellanes-Lozada, Cristina Cuautli, Natalya V. Likhanova, Marichel Desión-Palacios, Irina V. Lijanova, Janette Arriola-Morales, Octavio Olivares-Xometl

**Affiliations:** † Benemérita Universidad Autónoma de Puebla, Facultad de Ingeniería Química, Av. San Claudio y 18 Sur, Ciudad Universitaria. Col. San Manuel, Puebla, Puebla 72570, México; ‡ Dirección de Investigación, Instituto Mexicano del Petróleo, Eje Central Norte Lázaro Cárdenas No. 152, col. San Bartolo Atepehuacan, G. A. Madero, CDMX 07730, México; § Instituto Politécnico Nacional, CIITEC, Cerrada Cecati S/N, Colonia Santa Catarina de Azcapotzalco, CDMX CP 02250, México

## Abstract

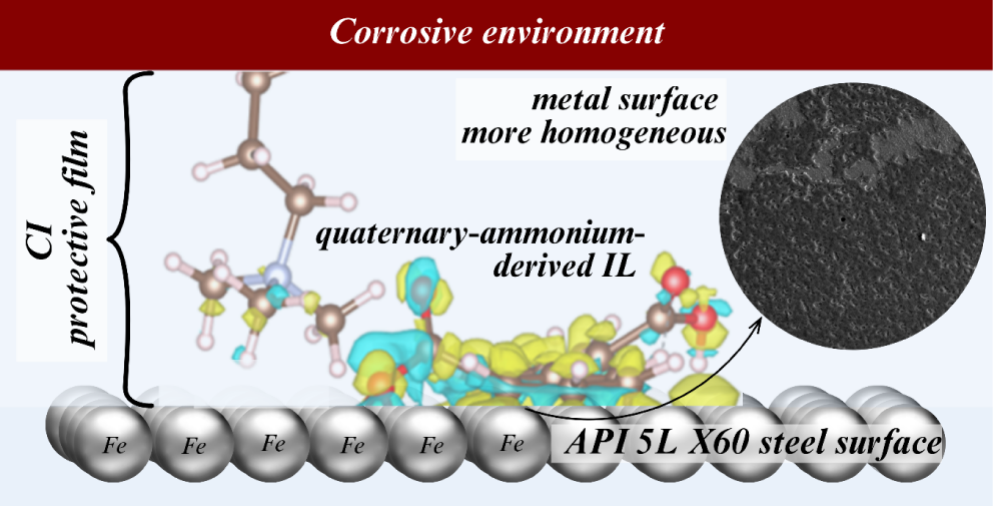

The properties of the ionic liquid (IL) trimethyldodecyl(tetradecyl)ammonium diphenyl-2,2’-dicarboxylate (C_12–14_TC_1_AmD) as a corrosion inhibitor (CI) of API 5L X60 steel in 1 M H_2_SO_4_ were studied at 298, 308, and 318 K. The inhibition efficiency (IE, %) was established by means of electrochemical techniques. The analysis confirmed that C_12–14_TC_1_AmD inhibited the corrosion process with a maximal IE of 90 ± 6% at 150 ppm. The inhibitor chemical configuration was a key factor in its physical (cation) and chemical (anion) adsorption on the steel surface, forming a protective film; due to the foregoing, the IL was classified as a mixed-type CI. Finally, the X-ray photoelectron spectroscopy (XPS) analysis confirmed the presence of IL on the metal surface, and scanning electron microscopy (SEM) evidenced less surface damage.

## 1 Introduction

In the field devoted to studying the corrosion phenomenon, it is known that the use of corrosion inhibitors (CIs) is one of the most efficient and inexpensive methods for controlling it.^1^ Its implementation requires the previous analysis of the conditions under which a CI will be working in order to be efficient. For instance, during downhole corrosion control, the application and dosing of a given CI is a function of the well conditioning and operation processes: well injection for protecting the inner wall of API carbon steel pipes; tubing injection mixed with acid solutions during the acidification stage to stimulate or clean the pipes; and the injection into the water-based fluid employed to fill the annular space between the production tubing and its outer casing during the well completion stage.^2,3^ Specifically, during the acidification stage, the operation conditions involve a temperature increase that requires the CI chemical stability so that it can preserve its effectivity.^4,5^ Different studies have associated the remarkable performance of CIs at high temperatures with a chemical adsorption mechanism through which the molecule shares either conjugated bonds or π*-electrons with the metal surface, thus forming strong chemical bonds; as a consequence, the adverse system temperature effects are not harsh enough for provoking the desorption of the CI, thus maintaining the desired inhibition efficiency (IE).

It is well known that organic compounds featuring polar headgroups with heteroatoms, aromatic rings, double and triple bonds, and long alkyl chains, among others, promote the chemical adsorption of the CI on the metal surface, thus reducing corrosion.^3,6−11^ Notwithstanding, in reality, the presence of these elements in the chemical structure of a molecule is not enough to work efficiently. Two molecules with the same number of atoms and functional groups but with dissimilar positions of their pendant groups can display different IEs.^12−14^ Even Wang et al., in 2023, stated that once a functional group, benzene ring, is adsorbed on the metal surface through chemical bonds, it provokes the electron density change of other molecule heteroatoms, thus modifying the subsequent interactions between the metal and the inhibitor.^1^ For this reason, the study of CIs with high-electron-density adsorption sites for efficient corrosion inhibition is not enough; it is necessary that the CI adsorption mechanism be established suitably to understand the scope that a certain molecule can have.

From the wide variety of chemical products evaluated as CIs to date, the quaternary-ammonium-derived compounds have shown to be highly efficient, slightly toxic, and relatively simple to synthesize.^15,16^ Wang et al., in 2023, evaluated three Mannich base imidazoline quaternary ammonium salts as CIs and stated that the formation of chemical bonds between quaternary ammonium derivatives and electrons from the empty 3d-orbital of iron atoms is possible, thanks to the pairs of free electrons in N and O atoms, as well as to delocalized conjugated π*-electrons.^11^ Other authors have analyzed the effect of pendant groups, which is the case in the study conducted by Neelam et al., in 2024, where it was indicated that the length of the alkyl chain in quaternary ammonium derivatives plays a major role in the inhibition process.^3^ The importance of the concentration in the formation of a protective homogeneous layer has also been analyzed, which is the case of Wang et al., in 2023, who evaluated the inhibiting properties of tetradecyl-benzyldimethylammonium bromide using TOF-SIMS and XPS and showed that at concentrations below 25 ppm, it is not possible to achieve the adequate CI adsorption that could favor the formation of a compact and continuous 0.15 nm layer.^1^ Likewise, Alahiane et al. developed various research works on benzoic acid derivatives against corrosion in hydrochloric acid medium, and based on the results from electrochemical and weight loss tests and theoretical calculations, the authors came to the conclusion that with the increasing number of hydroxy substituents in the benzoic ring, the corrosion inhibition efficiency is boosted.^17^

In the last decades, the study of ionic liquids (ILs) as CIs has wound up, and since they are considered as green compounds, they can comply with international regulations focused on the reduction of emissions of toxic residues into the environment; further advantageous features of these compounds are their high thermal stability and remarkable efficiency in reducing the metal corrosion rate.^12,16,18^ The design of ILs to be applied as CIs normally involves anions and cations of organic type that have diverse adsorption sites.^18^Table 1 shows some ILs with ammonium as the cation and organic anions such as those derived from the benzyl/carboxylate group evaluated as CIs of diverse metal types in different corrosive media, reaching IEs from 72 to 98% as a function of the inhibitor concentration (*C*_INH_).

**Table 1:**
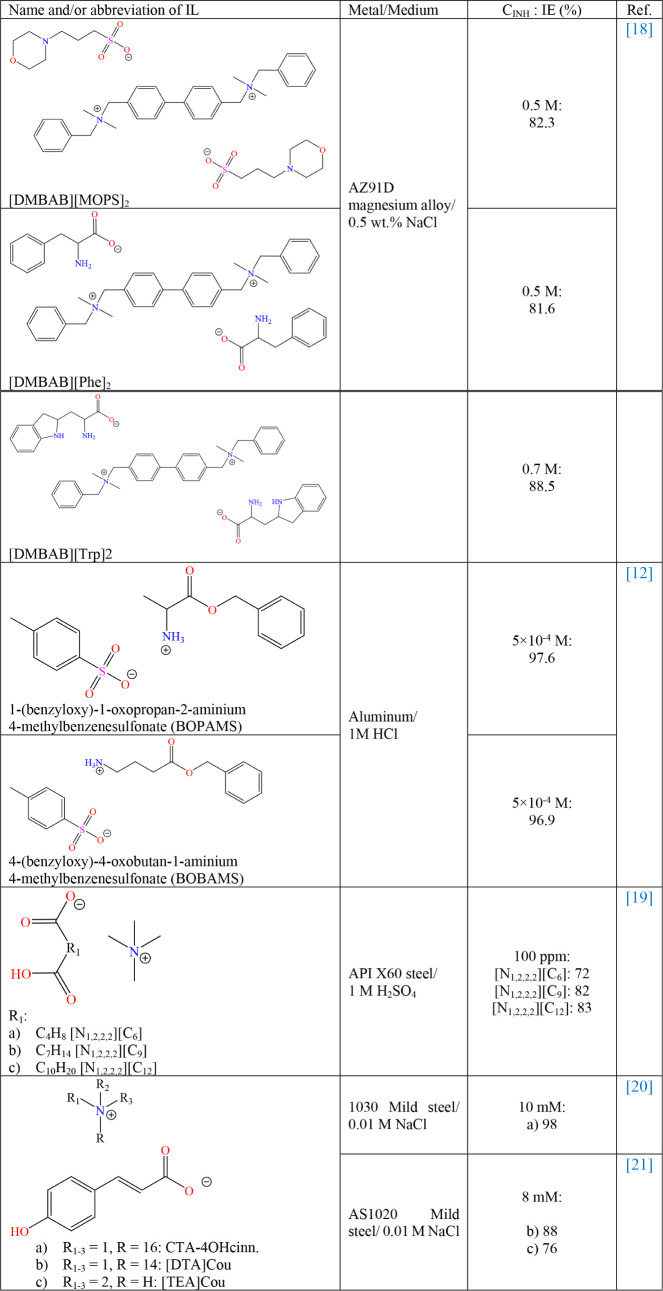
Chemical Structure of ILs with Ammonium as the Cation and Organic Anions Evaluated as CIs of Several Metal Types in Different Corrosive Media

Gao et al., in 2023, evaluated quaternary-ammonium-derived ILs featuring aromatic rings and carboxylic groups. The authors indicated that the synergy among the different adsorption sites (e.g., benzene ring, −NR_2_ (R=H or CH_3_), C=O) was possible, whereas the aromatic rings could be adsorbed by backdonation of π*-electrons, and the quaternary ammonium group was adsorbed on the metal surface by electrostatic interactions.^18^ Likewise, Nesane et al., in 2020, investigated the anticorrosive effect on aluminum at different temperatures by employing two ILs based on quaternary ammonium with aromatic rings, double bonds, and other heteroatoms (BOPAMS and BOBAMS). The authors indicated that the adsorption of BOPAMS/BOBAMS could be carried out in one to three steps: electrostatic interactions between the charged metal surface and BOPAMS/BOBAMS; the formation of coordinate bonds by the CI unshared electron pairs and the empty or half-filled p-orbitals of Al atoms; and the participation of CI π*-electrons.^12^ On the other hand, a study carried out in 2020 by our research group dealt with the analysis of ILs derived from quaternary ammonium and carboxylic acids, where it was concluded that the IL with the anion dodecanedioate, a type of carboxylic acid, presented the best IEs.^19^ Other ammonium-based structures, but with anions derived from the benzyl/carboxylate group, were studied by Soto-Puelles et al.^20^ and Monaci et al.^21^ In the first study, the authors suggested that the pitting process was slowed by the formation of an organic film on the metal surface, where the carboxylate groups interacted by means of their electronic charge delocalized on the two oxygen atoms, whereas the C=C bond stabilized the process. Notwithstanding, Monaci et al. pointed out the synergistic effect between the IL cation and anion during the inhibition process and suggested that the alkyl chain in both parts of the IL played a major role in the stability during the immersion of the metal samples, boosting the inhibiting effect with longer alkyl chains. The latter led us to think that an IL with a cation derived from quaternary ammonium with a 14-carbon-atom alkyl chain and an anion derived from dicarboxylic acid with aromatic rings could present better inhibiting behavior, favored by the formation of chemical bonds between the IL and the metal surface. According to the foregoing, the present study emphasized the importance of the chemical configuration of the cation and the anion in IL C_12–14_TC_1_AmD as a CI of API 5L X60 steel in 1 M H_2_SO_4_. Furthermore, an IL whose chemical structure is free of halogens and IE increases with temperature is presented. The corrosion rate, inhibitor type, and electrochemical behavior were defined by means of the potentiodynamic polarization (PDP) and electrochemical impedance spectroscopy (EIS) techniques. The morphology and chemical state of the elements at the metallic interface were studied by scanning electron microscopy (SEM) and X-ray photoelectron spectroscopy (XPS). Electronic structure calculations were carried out under the framework of density functional theory (DFT).

## 2 Experimental Section

### 2.1 Synthesis and Characterization of the CI

The compound ADMA 1214 AMINE was purchased from Albemarle Corporation, diphenic acid (97%) and methanol were purchased from Merck, and dimethyl carbonate (99%) was purchased from Sigma-Aldrich.

The compound was characterized by ^1^H NMR and ^13^C NMR, and spectra were recorded on a JEOL Eclipse-300 equipment in CDCl_3_; chemical shifts were expressed in ppm relative to tetramethylsilane as an internal standard. The compound was synthesized in two steps: 1. synthesis of trimethyldodecyl(tetradecyl)ammonium methyl carbonate and 2. anion exchange with the formation of trimethyldodecyl(tetradecyl)ammonium diphenyl-2,2’-dicarboxylate. Trimethyldodecyl(tetradecyl)ammonium methyl carbonate was synthesized similarly to a previously reported methodology.^22^

Trimethyldodecyl(tetradecyl)ammonium methyl carbonate (C_12–14_TC_1_AmMC): 11.15 g of ADMA 1214 AMINE (50 mmol) reacted with 9.01 g (100 mmol) of dimethyl carbonate in 20 mL of methanol. The synthesis was performed using a Parr reactor model 4848, equipped with a stirrer and temperature controls, and the reaction mixture was kept at 423 K under autogenous pressure and stirring for 6 h.

Once the reaction finished, the mixture was filtered, and the residual dimethyl carbonate and methanol were evaporated under vacuum. The crude compound was washed three times with ethyl acetate (3 × 50 mL). Finally, the solvents were removed with a vacuum. 14.40 g of a yellow viscous liquid were obtained with a yield of 92%. ^1^H NMR (CDCl_3_): δ 0.94 (t, *J* = 6 Hz, 3H), 1.31–1.40 (m, 18H), 1.75–1.79 (m, 2H), 3.34 (s, 9H), 3.41 (s, 3H), 3.45–3.48 (m, 2H) ppm. ^13^C NMR (CDCl_3_): δ 13.8 (1C), 22.4 (1C), 22.8 (1C), 25.9 (1C), 28.9 (1C), 29.0 (1C), 29.1 (1C), 29.2 (1C), 29.3 (1C), 29.4 (1C), 31.6 (1C), 52.0 (1C), 52.6 (3C), 66.3 (1C), and 158.1 (1C) ppm.

Trimethyldodecyl(tetradecyl)ammonium diphenyl-2,2’-dicarboxylate (C_12–14_TC_1_AmD): 4.84 g of diphenic acid (20 mmol) was dissolved in 25 mL of methanol, and 25 mL of methanol solution containing 6.26 g (20 mmol) of C_12–14_TC_1_AmMC was added to the mixture. The reaction occurred with the release of gas and was kept under constant stirring for 0.5 h at 337 K. After reaction completion, the solvent was removed by vacuum evaporation, and 9.10 g of raw product with a yield of 95 % was obtained. The synthesized IL was recrystallized from an ethanol/ethyl acetate (10:1) mixture, filtered, and dried under vacuum; finally, a yellowish crystalline compound was obtained. M.p. 322 K. ^1^H NMR (CDCl_3_): δ 0.86 (t, *J* = 6 Hz, 3H), 1.15–1.28 (m, 18H), 1.52 (m, 2H), 2.86 (s, 9H), 3.08 (m, 2 H), 7.02 (dd, *J*_1_ = 6, *J*_2_ = 0.06 Hz, 2H), 7.28–7.34 (m, 4H), 7.58 (dd, *J*_1_ = 6, *J*_2_ = 0.06 Hz, 2H) ppm. ^13^C NMR (CDCl_3_): δ 13.9 (1C), 22.4 (1C), 22.7 (1C), 25.8 (1C), 28.9 (1C), 29.0 (1C), 29.1 (1C), 29.2 (1C), 29.3 (1C), 29.4 (1C), 31.6 (1C), 52.6 (3C), 66.6 (1C), 127.2 (2C), 127.3 (2C), 128.7 (2C), 129.4 (2C), 136.3 (2C), 139.1 (2C), and 173.7 (2C) ppm.

### 2.2 Materials and Preparation of Solutions

API 5L X60 steel coupons were employed as metallic substrates, and their composition (wt %) was as follows: C 0.12, Si 0.45, Mn 1.60, P 0.025, S 0.015, V 0.05, Nb 0.05, Ti 0.04, with Fe as the balance. The steel samples were prepared prior to each measurement according to the following procedure: first, the surface was abraded by using SiC abrasive paper with grit sizes of 600, 800, 1000, and 1200; after the abrading process, the samples were rinsed with isopropanol and acetone; finally, they were dried with dry air. It is worth mentioning that only for the surface analysis tests, after the abrading process, the metal surface was polished with 1-μm alumina until achieving mirror finishing. The corrosive medium was a 1 M H_2_SO_4_ aqueous solution, which was prepared by using deionized water and analytical-grade sulfuric acid. Dilutions of 25, 50, 75, 100, and 150 ppm of C_12–14_TC_1_AmD in ethanol were used in the corrosive medium to carry out the electrochemical tests; in the case of the surface analyses, only the concentration at 150 ppm was evaluated.

### 2.3 Electrochemical Techniques

The electrochemical experiments were performed by means of a potentiostat/galvanostat coupled with a computer controlled by the software NOVA version 2.1.4. The employed techniques were PDP and EIS.^23−25^ The potentiostat was connected to three electrodes adapted for a glass electrochemical cell. An Ag/AgCl electrode was used as a reference electrode along with a platinum counter electrode (99.9%) and API 5L X60 steel, with an exposed surface area of 0.1779 cm^2^, as a working electrode. The cell was adapted to a temperature recirculation system to carry out corrosion tests at 298, 308, and 320 K. Before each test, the open circuit potential (*E*_OCP_) was monitored for 900 s. The PDP experiments were performed by considering an interval ranging from −250 to +250 mV, with respect to the obtained *E*_OCP_, at a scanning rate of 0.166 mV s^–1^. As for the EIS tests, they were carried out at frequencies from 100 kHz to 10 mHz under *E*_OCP_ conditions, applying AC with a signal amplitude perturbation of 5 mV; the software NOVA version 2.1.4 was used to fit the experimental data to electrical equivalent circuits (EECs). In order to ensure the reproducibility of results, the electrochemical tests were done in triplicate.^26^

### 2.4 Surface Analysis Techniques

The steel samples were analyzed by SEM and XPS after being immersed in 1 M H_2_SO_4_ with and without adding 150 ppm of C_12–14_TC_1_AmD for 4 h.^23,27^ The morphological analysis was carried out by means of a JEOL-JSM-6300 SEM piece of equipment. The chemical state of the elements present on the metal surface was established by the XPS technique using a K-alfa spectrometer with a monochromatic X-ray source and Al anode; the analyzer pass energy was 20 eV. All the obtained spectra were referred to the signal at 284.8 eV, corresponding to adventitious carbon. The analysis of the general spectrum and deconvolution of the high-resolution spectra was accomplished by means of the software CASPA XPS version 2.3.25PR1.0.

### 2.5 Molecular Simulation

The interaction between the IL C_12–14_TC_1_AmD and API X60 steel was analyzed by means of first-principles calculations within the density functional theory. Periodic calculations were performed to solve the Kohn–Sham equations, imposing periodic boundary conditions as implemented in the Siesta 4.1 code.^28^ The Perdew–Burke–Ernzerhof (PBE)^29^ functional was used for the correlation-exchange term, and dispersion corrections were considered through the Grimme scheme.^30^ Sankey and Niklewsky PAO basis^31^ was used along with pseudopotentials obtained from the National Nanotechnology Infrastructure Network.^32^ Full geometric optimizations were carried out through the conjugate gradient method. The convergence criteria were 1 × 10^–4^ eV and 0.1 eV/Å for energy and force, respectively. The interaction energy was calculated by employing eq 1:
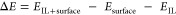
1where *E*_IL+surface_ corresponds to the energy of the ions adsorbed on the Fe surface, while *E*_surface_ and *E*_IL_ represent the energy of the clean surface and cation–anion complex, respectively. The basis set superposition error (BSSE) corrections were considered through the geometrical counterpoise scheme.^33^ Fukui functions for the isolated ions were calculated in the gas phase, considering the geometry of the adsorbed IL. Single-point calculations at the PBE0^34^-D3^35,36^/def2-TZVP^37^ level of theory were carried out as implemented in the Orca 5.0.1 code.^38^ The steel surface was modeled through a surface slab of Fe [110], and the model thickness was set to 4 iron layers determined for convergence studies. The distance between slabs was ∼33.8 Å. The cation adsorption was proposed by approaching the anion to the surface, so the position of both rings is favored and is as similar as possible to the most stable position observed for benzene adsorbed on the Fe [110] surface. The cation was positioned near the anion. After the construction, geometry optimization and interaction analysis were carried out to describe the adsorption mechanism.

## 3 Results and Discussion

### 3.1 Electrochemical Tests

**Figure 1:**
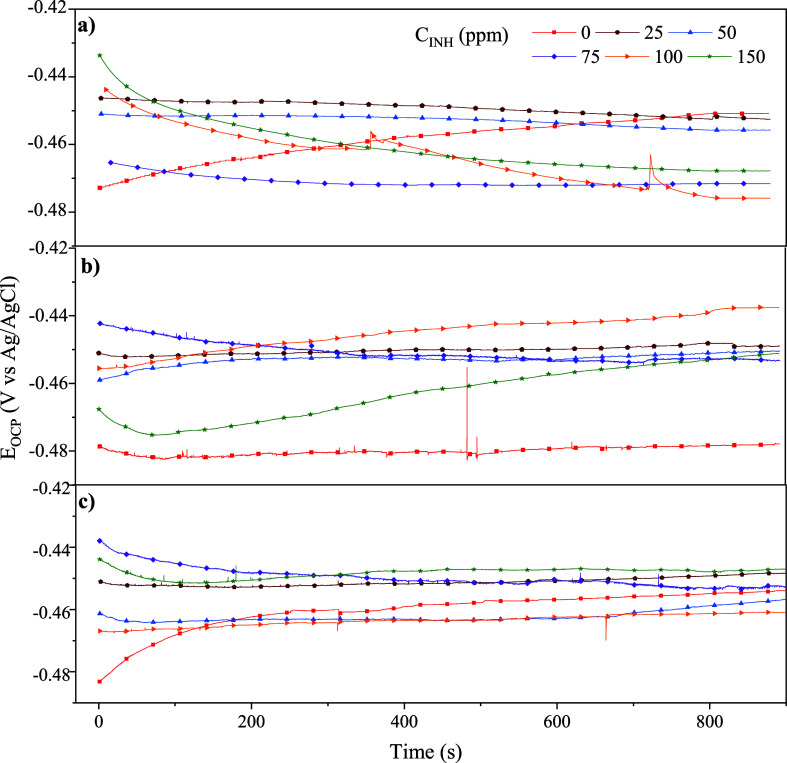
OCP variation with time for API 5L X60 steel in 1 M H_2_SO_4_ containing C_12–14_TC1AmD at (a) 298 K, (b) 308 K, and (c) 318 K.

Figure 1 shows the *E*_OCP_ measurement as a function of the immersion time of API 5L X60 steel in corrosive systems with different C_12–14_TC_1_AmD concentrations and temperatures. It can be observed that all of the curves approach a constant potential with a stable behavior pattern after 900 s of immersion. Additionally, at all the temperatures, an *E*_OCP_ displacement of the systems with IL with respect to the blanks is shown, which indicates that the presence of C_12–14_TC_1_AmD modifies the state of the thermodynamic equilibrium of the electrochemical semireactions.

**Figure 2:**
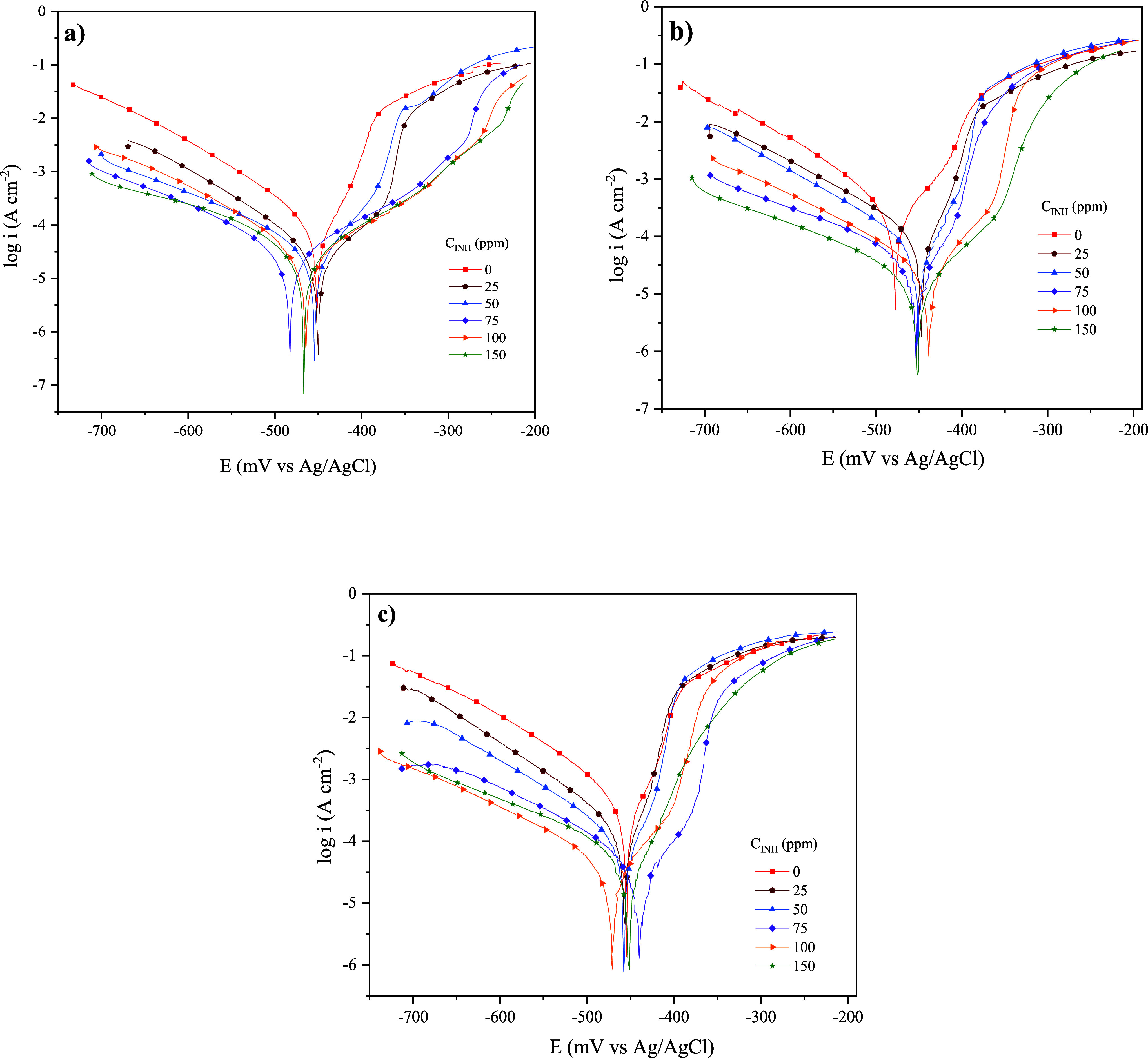
Tafel curves of API 5L X60 steel in 1 M H_2_SO_4_ with and without C_12–14_TC_1_AmD obtained by the PDP technique at (a) 298 K, (b) 308 K, and (c) 318 K.

Figure 2 shows the Tafel curves of API 5L X60 steel in 1 M H_2_SO_4_ in the absence and presence of CI at different temperatures. It is observed that all of the curves in the presence of C_12–14_TC_1_AmD feature a current density displacement (*i*) toward values that are lower than those of the blank. Since *i* has a directly proportional relationship with the corrosion rate, it can be inferred that the compound addition to H_2_SO_4_ reduces the corrosion rate of API 5L X60 steel. Furthermore, the cathodic Tafel curves display a behavior pattern that is characteristic of a corrosion process controlled by activation.^39^ In the systems with C_12–14_TC_1_AmD addition, the cathodic branches are practically parallel to the curves without inhibitor and are displaced toward lower current density values, indicating that the addition of CI to the acid solution does not modify the mechanism of hydrogen evolution and reduction of H^+^ on the steel surface but retards the reduction reaction rate of the metal–medium system through a molecular adsorption process on cathodic sites of the metal surface. As for the anodic Tafel curves, two different potential polarization regions marked by a change in the anodic slope can be seen: region I is close to the corrosion potential (*E*_corr_) and region II is close to more positive potential values. With the increasing CI concentration in region I, an evident displacement of the curves toward lower current density values can be observed, suggesting the adsorption of C_12–14_TC_1_AmD molecules on anodic sites, i.e., in zones where the dissolution of Fe takes place, and then the anodic reaction rate is reduced. In all the corrosive systems, between regions I and II, a slope change occurred, starting at a potential known as the anodic desorption potential of the CI.^11^ It is observed that with increasing concentration, such potential is more positive (Figure 2), suggesting that at higher amounts of C_12–14_TC_1_AmD molecules in the corrosive medium, the formation of a stable protecting film, allowed by the CI adsorption, is possible; this fact became more evident at 298 K (Figure 2a). Likewise, the change observed in the anodic branch within the interval from −400 to −300 mV can suggest the possible desorption of the CI and/or the formation of intermediate iron species with corrosive medium ions and a film of corrosive products, which as a whole limits the diffusion of species and interrupts the prolonged attack of the metal surface.^40^

The application of a high anodic polarization to the steel material provokes the accelerated dissolution of iron, even in the presence of highly efficient CIs, as reported by diverse authors.^11,41^ The CI desorption process is not an instantaneous phenomenon, as there is a transition stage at which, while some molecules are adsorbed on the metal substrate, others are desorbed, and when the adsorption rate is lower than the desorption rate, the metal surface ends up exposed and vulnerable to the corrosion process. This fact can be observed in region II, at more positive potential values, where the current density values of the polarization curves in the presence of inhibitor approach the blank systems, indicating the partial desorption of the CI. It is worth emphasizing that this phenomenon did not occur at 298 K, suggesting the electrochemical stability of C_12–14_TC_1_AmD. Since the curves in Figure 2 show an anodic region that is far from the characteristic Tafel behavior, i.e., they do not display a linear trend for at least a current decade, the electrochemical parameters were established from the intersection of the linear extrapolation of the cathodic curve and *E*_OCP_.^39,42,43^Table 2 presents the *E*_corr_, corrosion current density (*i*_corr_), cathodic Tafel slope (β_c_), and PDP (IE_PDP_) inhibition efficiency values. The IE_PDP_ values were obtained by employing the following equation:
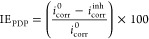
2where  and  are inhibited and uninhibited corrosion current densities, respectively.

**Table 2:** Electrochemical Parameters of API 5L X60 Steel in 1 M H_2_SO_4_ with C_12–14_TC_1_AmD at Different Temperatures Obtained by PDP

*T* (K)	*C* (ppm/mM)	–*E*_corr_ (mV)	Δ*E*_corr_	–β_c_ (mV/dec)	*i*_corr_ (μA/cm^–2^)	IE_PDP_ (%)
298	0/0	458 ± 6		95 ± 2	584 ± 7	-
	25/0.0522	450 ± 5	–8	100 ± 7	311 ± 17	47 ± 2.9
	50/0.1043	453 ± 3	–5	89 ± 1	193 ± 14	67 ± 2.4
	75/0.1565	472 ± 2	14	121 ± 6	136 ± 14	77 ± 2.4
	100/0.2086	481 ± 7	23	115 ± 2	125 ± 14	79 ± 2.4
	150/0.3130	466 ± 4	8	141 ± 4	101 ± 3	83 ± 0.6
308	0/0	475 ± 5		97 ± 2	1079 ± 9	-
	25/0.0522	447 ± 6	–28	103 ± 5	520 ± 14	52 ± 1.4
	50/0.1043	450 ± 3	–25	112 ± 2	253 ± 8	77 ± 0.8
	75/0.1565	460 ± 9	–15	108 ± 4	185 ± 7	83 ± 0.7
	100/0.2086	440 ± 2	–35	118 ± 4	112 ± 7	90 ± 0.7
	150/0.3130	455 ± 3	–20	160 ± 6	73 ± 8	93 ± 0.7
318	0/0	458 ± 6		100 ± 2	1809 ± 3	-
	25/0.0522	458 ± 5	0	101 ± 2	668 ± 9	63 ± 0.5
	50/0.1043	453 ± 8	–5	102 ± 6	327 ± 10	82 ± 0.6
	75/0.1565	450 ± 4	–8	126 ± 5	254 ± 10	86 ± 0.6
	100/0.2086	463 ± 3	5	124 ± 8	171 ± 2	91 ± 0.1
	150/0.3130	442 ± 7	–16	130 ± 9	98 ± 12	95 ± 0.7

**Figure 3:**
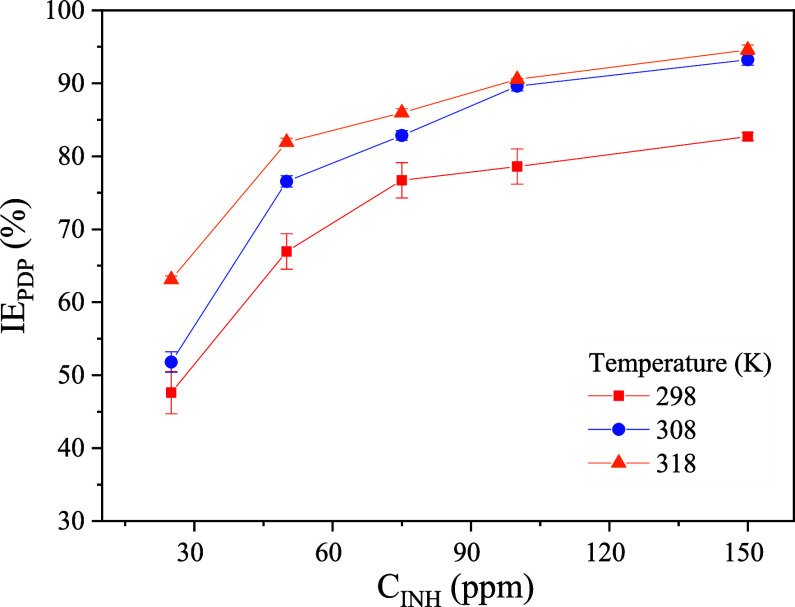
IE_PDP_ of API 5L X60 steel in 1 M H_2_SO_4_ in the absence and presence of C_12–14_TC_1_AmD.

The *E*_corr_ displacements shown in Table 2, calculated through the *E*_corr_ difference between the system with and without CI () do not follow a specific anodic or cathodic trend, positive or negative values, respectively, and are not greater than ±85 mV, suggesting that C_12–14_TC_1_AmD works as a mixed-type inhibitor.^43^ C_12–14_TC_1_AmD is adsorbed on the metal surface through geometric blockage, reducing equally the active sites where the iron dissolution reactions and hydrogen evolution take place. The *i*_corr_ values of the systems without C_12–14_TC_1_AmD increased with the temperature, as was expected, as higher temperatures boosted the system’s internal energy and, with it, the rate of the involved electrochemical reactions. The addition of C_12–14_TC_1_AmD to the corrosive medium caused, at any temperature, a diminution of the *i*_corr_ values and then a reduction of the iron degradation even with the increasing temperature. These results are in good agreement with the IE_PDP_ values, see Table 2 and Figure 3, where it is observed that the increase in concentration and temperature provoked a growing pattern in the inhibition efficiency of C_12–14_TC_1_AmD.

The maximal IE_PDP_ value obtained with 150 ppm of C_12–14_TC_1_AmD in 1 M H_2_SO_4_ at 318 K was 95%. Different research works evaluating CIs have stated that molecular desorption from the metal surface occurs with increasing temperature and have associated it with physical-type CI adsorption. In contrast, other authors have suggested that the IE increase is possible with higher temperatures when there is a formation of chemical bonds between the inhibitor molecule and the metal surface.^44^ The latter could reveal that higher IE values at 318 K are due to a chemical adsorption process between C_12–14_TC_1_AmD and the steel surface; however, the results of further techniques will help clarify this fact.

**Figure 4:**
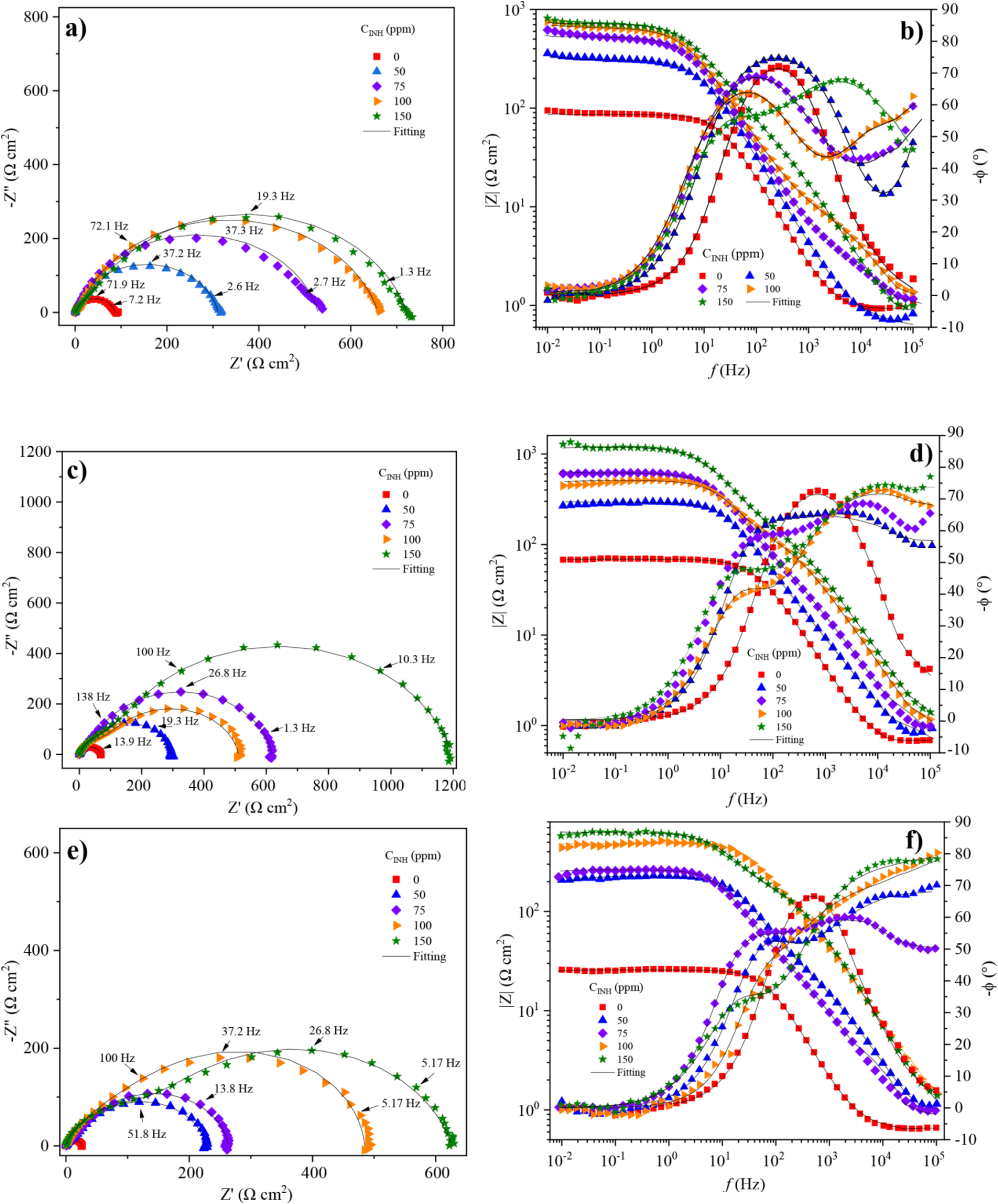
Impedance spectra of API 5L X60 steel in 1 M H_2_SO_4_ in the absence and presence of C_12–14_TC_1_AmD: (a) Nyquist – 298 K, (b) Bode – 298 K, (c) Nyquist – 308 K, (d) Bode – 308 K, (e) Nyquist – 318 K, and (f) Bode – 318 K.

Figure 4a–f displays the Nyquist and Bode impedance curves of API 5L X60 steel in 1 M H_2_SO_4_ in the absence and presence of CI at 298, 308, and 318 K. In the Nyquist plots, Figure 4a,c,e, it is observed that the systems without and with low C_12–14_TC_1_AmD concentrations feature spectra with the capacitive reactance arc shape that is characteristic of steel corrosion processes; furthermore, with the increasing CI concentration, the shape of the spectra is slightly modified, observing two capacitive reactance semiarcs instead of just one. All of the semicircles are depreciated, indicating the surface heterogeneity of the steel samples. Additionally, at all the temperatures, the presence of C_12–14_TC_1_AmD provoked a diameter increase in the capacitive reactance arcs as a function of the concentration, showing that the addition of C_12––14_TC_1_AmD produced an increase in the involved resistances in the metal–solution interface, which confirms that the addition of the IL to the metal surface modifies the electron transfer rate and then the kinetics of the electrochemical reactions. The Bode phase plots shown in Figure 4b,d, f, in the absence of CI, display a curve with a maximal point at approximately 70°, whereas the impedance module Bode curves feature a linear behavior pattern at frequencies between approximately 5 × 10^1^ and 5 × 10^3^ Hz. The shape of these spectra is characteristic of carbon steel systems in sulfuric acid medium and reveals the presence of a time constant that is related as well to a constant phase element (CPE) connected in series with one electrical resistance (*R*).^45^ In contrast, at 150 ppm of C_12–14_TC_1_AmD, the phase Bode curves present two maximal phase points at approximately 2 × 10^1^ Hz and 8 × 10^3^ to 1 × 10^4^ Hz, indicating two time constants related to two CPEs and two Rs. As for systems with intermediate concentrations, a remarkable transition between one and two time constants can be observed in the phase Bode plot, Figure 4b,d,f, from a wider peak, with respect to the blank (for example, at 50 ppm and 298 K) to the evident presence of two maximal points. These results indicate that the addition of C_12–14_TC_1_AmD modified the electrical behavior at the metal interface.

**Figure 5:**
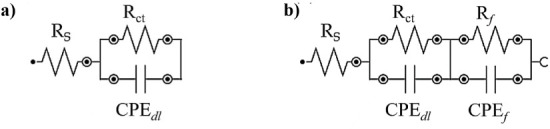
EEC for fitting EIS experimental data of API 5L X60 steel in 1 M H_2_SO_4_: (a) absence and (b) presence of C_12–14_TC_1_AmD.

The calculation of the electrochemical parameters by the EIS technique was carried out by fitting the experimental data to an EEC, as shown in Figure 5.

Due to the surface heterogeneity of the steel samples, a CPE was employed instead of an ideal capacitance. The electrical elements that are part of the EEC are described as follows: *R*_s_ is the resistance to the solution, *R*_ct_ is the resistance to the charge transfer on the surface and depends on the charge transfer between the electronic conduction region (metal) and electrolyte, CPE_dl_ is the constant phase element associated with the electrical double layer and describes the charge accumulation at the metal–solution interface, and CPE_f_ and *R*_f_ are the constant phase element and resistance related to the adsorption of C_12–14_TC_1_AmD molecules on the metal surface, respectively.^46^ The calculation of the pseudocapacitance or nonideal capacitance was performed with the following equation:
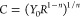
3where *Y*_0_ is the proportional factor and *n* is the empirical exponent between 0 and 1, which is related to the heterogeneity of the surface. The values obtained from fitting of the EECs are reported in Table 3.

**Table 3:** EIS Parameters Obtained for API 5L X60 Steel in 1 M H_2_SO_4_ with C_12–14_TC_1_AmD at Different Temperatures

*T* (K)	*C* (ppm/mM)	*R*_s_ (Ω cm^2^)	*R*_ct_ (Ω cm^2^)	*C*_dl_ (μF cm^–2^)	*R*_f_ (Ω cm^2^)	*C*_f_ (μF cm^–2^)	χ^2^	Rp_EIS_ (Ω cm^2^)	IE_EIS_ (%)
298	0	0.84 ± 5	86 ± 5	91.62 ± 2			0.036	86 ± 5	-
	50/0.1043	1 ± 1	346± 1	66.3 ± 2.3	8 ± 0.23	14.73 ± 0.02	0.054	355 ± 1	75 ± 1.4
	75/0.1565	0.23 ± 0.43	541 ± 2	59.0 ± 2.0	15 ± 0.31	4.46 ± 0.03	0.032	556 ± 2	84 ± 0.9
	100/0.2086	0.22 ± 0.21	652 ± 1	56.3 ± 3.0	26 ± 0.42	5.04 ± 0.07	0.036	678 ± 1	87 ± 0.7
	150/0.3130	1 ± 2	703 ± 1	43.4 ± 3.0	123 ± 1	22.25 ± 3.01	0.050	825 ± 1	89 ± 0.6
308	0	0.50 ± 4	68 ± 4	50.25 ± 3			0.019	68 ± 4	-
	50/0.1043	0.12 ± 0.04	283 ± 0.4	50.4 ± 1.0	15 ± 1	71.39 ± 0.004	0.031	298 ± 0.4	77 ± 1.3
	75/0.1565	0.23 ± 0.10	531 ± 0.1	37.9 ± 1.9	106 ± 2	22.15 ± 0.004	0.030	637 ± 0.1	89 ± 0.6
	100/0.2086	0.19 ± 0.01	581 ± 8	39.8 ± 2.0	38 ± 1	7.51 ± 1.00	0.023	619 ± 8	89 ± 0.7
	150/0.3130	0.01 ± 0.01	1001 ± 2	28.3 ± 6.2	104 ± 0.33	6.6 ± 0.05	0.036	1105 ± 2	94 ± 0.4
318	0	0.56 ± 11	25 ± 5	99.49 ± 2			0.058	25 ± 5	-
	50/0.1043	0.26 ± 0.43	229 ± 1	42.6 ± 1.9	15 ± 1	14.53 ± 0.14	0.019	244 ± 1	89 ± 2.0
	75/0.1565	0.14 ± 0.21	280 ± 1	35.8 ± 2.5	40 ± 1	20.57 ± 0.02	0.027	320 ± 1	92 ± 1.5
	100/0.2086	0.17 ± 0.25	450 ± 0.3	39.2 ± 0.5	99 ± 2	8.4 ± 0.14	0.127	548 ± 0.3	95 ± 0.9
	150/0.3130	0.06 ± 0.02	538 ± 2	36.4 ± 1.0	140 ± 1	4.59 ± 0.04	0.064	678 ± 2	96 ± 0.7

The suitable methodological process for carrying out the electrochemical experiments was revealed by the low *R*_s_ values, which indicated that the ohmic resistance was minimal in all of the corrosive systems. As for the values corresponding to *R*_ct_ and pseudocapacitance of the electrical double layer (*C*_dl_), it is observed that they increased and diminished with the C_12–14_TC_1_AmD concentration, respectively. The latter suggests that the resistance to the charge transfer is increased by the formation of a protecting layer consisting of C_12–14_TC_1_AmD molecules, which allowed a lower storage release of electrons/charge. The *R*_f_ and *C*_f_ values, in general, are much lower than those of *R*_ct_ and *C*_dl_, which confirms the modification of the electronic properties of the metal surface by the presence of adsorbed C_12–14_TC_1_AmD molecules. Different studies have reported that an additional capacitive electrical element to *C*_dl_ suggests that the CI adsorption allows the formation of an additional homogeneous layer with different thicknessess and dielectric properties.^6,16,47^ In this study, it is observed that despite the contribution of *R*_f_ and *C*_f_ being lower than that of *R*_ct_ and *C*_dl_, the presence of these electrical elements in the employed EECs indicates that C_12–14_TC_1_AmD is adsorbed homogeneously on the metal surface, generating the blockage of the active sites where the hydrogen evolution and iron dissolution reactions take place. Additionally, the inhibition efficiency established with the EIS (IE_EIS_) data is shown in Table 3 and was calculated with eq 4:
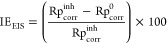
4where  and  are the polarization resistance with and without CI, respectively. The Rp values were obtained by adding *R*_ct_ and *R*_f_.^48^ The IE_EIS_ values are in accordance with those obtained by the PDP technique, which confirmed that the concentration and temperature increase improved the C_12–14_TC_1_AmD effectivity inhibiting the corrosion of API 5L X60 steel in an acid medium.

### 3.2 Kinetic and Thermodynamic Analysis

**Figure 6:**
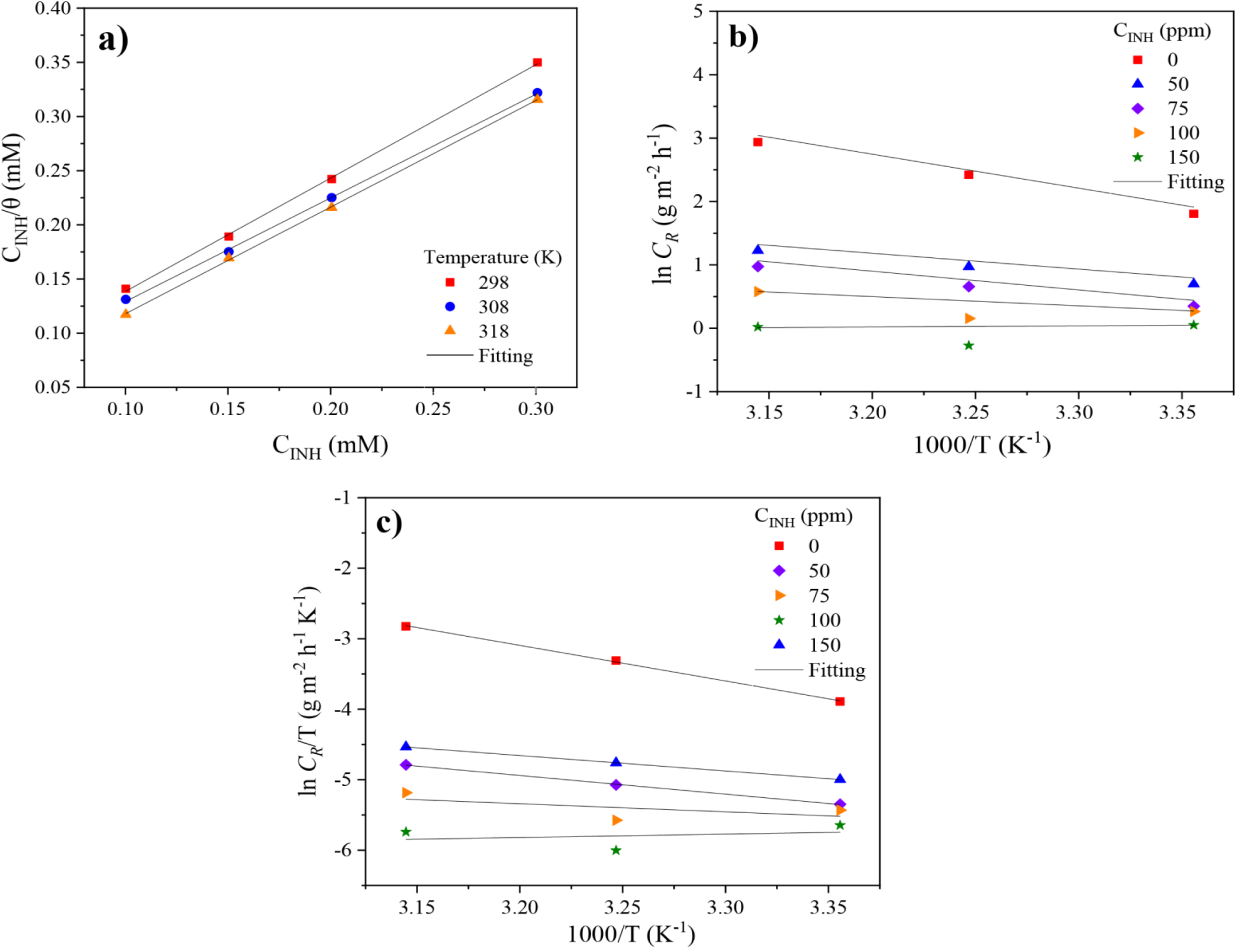
(a) Langmuir adsorption isotherm, (b) transition state, and (c) Arrhenius plot of API.

As discussed, the compound C_12–14_TC_1_AmD works as a CI through an adsorption process on the metal surface, where two mechanisms are possible: physisorption or chemisorption. One of the methods for clarifying the existing interaction between CI molecules (adsorbate) and a metallic substrate is the use of adsorption isotherms. In the area of CIs, different adsorption isotherm models have been employed to fit experimental results, with the mathematical models of Langmuir, Temkin, Frumkin, and Freundlich being the most used.^49^ In the calculation of adsorption isotherms, the surface coverage degree (θ) represents the metallic surface fraction protected by CI molecules and is a function of its concentration. In the present work, θ values were calculated with the average value between IE_PDP_ and IE_EIS_ by θ = IE/100.^50^Figure 6 presents the fitting of the experimental data to the modified Langmuir adsorption isotherm, whose linear expression is the following:^51,52^
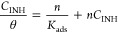
5where *C*_INH_ is the C_12–14_TC_1_AmD concentration, *K*_ads_ is the adsorption equilibrium constant, and *n* is related to the adsorption sites occupied by an inhibitor molecule.

The excellent fitting of the experimental data to the adsorption isotherm can be observed, whereas the *R*^2^ values equal to 0.999 (Table 4) indicate that the thermodynamic parameters obtained from the Langmuir model are highly reliable. The selection of this modified version of the original Langmuir equation was done because of the slight deviation of 1 of the slopes (Table 4), which is associated with interadsorbate interactions, multisite adsorption, or surface heterogeneity.^51^ Furthermore, at 308 and 318 K, *n* < 1 values indicate that the temperature increase provokes slight attractive interactions between C_12–14_TC_1_AmD molecules.^51^

**Table 4:** Thermodynamic Parameters of API 5L X60 Steel in 1 M H_2_SO_4_ with C_12–14_TC_1_AmD

*T* (K)	*R*^2^	*n*	*K*_ads_ (L mol^–1^)	(kJ mol^–1^)
298	0.9995	1.05	32326	35.7
308	0.9996	0.96	28799	36.6
318	0.9998	0.98	50193	39.2

Table 4 shows the *K*_ads_ values obtained from the *n*/intersection ratio of the *C*_INH_/θ versus *C*_INH_ plots. These high values evidence the strong inhibitor adsorption on the metal surface.^53^ On the other hand, the values of the standard Gibbs free energy of adsorption () (Table 4) were calculated through the following expression:^54^
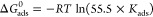
6where *R* is the ideal gas constant (8.314 × 10^–3^ kJ mol^–1^ K^–1^), *T* is the absolute temperature in K, and 55.5 is the concentration of water in mol L^–1^.^4,42^ It can be seen that the values are within the interval ranging from −40 to −20 kJ mol^–1^, i.e., it is not an exclusive physisorption or chemisorption process, which suggests that the adsorption process of C_12–14_TC_1_AmD on the steel surface in 1 M H_2_SO_4_ occurs through a physicochemical mechanism.^42,55,56^ According to different research works, the chemisorption process is related to the formation of covalent bonds between the vacant d*-*orbitals of the iron atoms and the free electron pairs present in oxygen heteroatoms, as well as to a backdonation process of electrons between steel and aromatic cycles.^18,43,57,58^ In the case of C_12–14_TC_1_AmD, these interactions could occur in aromatic rings and the anion carboxylic group. As for physical adsorption, it is possible by weak electrostatic interactions, as well as by Van der Waals bonds between the nitrogen heteroatom of the quaternary ammonium functional group in the cation of C_12–14_TC_1_AmD and the metal surface.^6,18,55,56,58^ Due to the fact that in the last years there has been controversy about how the interpretation of the  values establishes the adsorption mechanism of a CI,^51,59,60^ in the present work, additional computational modeling studies were carried out to consolidate the adsorption mechanism.

In order to analyze the temperature effect on the inhibition mechanism of C_12–14_TC_1_AmD against steel corrosion, the activation energy and thermodynamic parameters were determined from the PDP results. The activation energy (*E*_a_) of the corrosive media in the presence of IL was calculated with the Arrhenius equation (eq 7), employing the corrosion rate values *C*_R_ (eq 8).^11,15^ The results are shown in Figure 6 and Table 5:
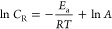
7
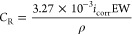
8where *A*, EW, and ρ are the preexponential factor, equivalent weight, and alloy density, respectively.^61^Table 5 shows that the *E*_a_ values diminish with the increasing C_12–14_TC_1_AmD amount from 44.6 J/mol in the absence of CI to 24.6 J/mol in the presence of 75 ppm of C_12–14_TC_1_AmD. This fact suggests that charge transfer occurs from the inhibitor to the metal surface to form coordinated covalent bonds, that is, through a chemisorption process.^15,62,63^ On the other hand, the standard enthalpy of adsorption  and standard entropy of adsorption  were calculated from the transition state expression according to the following eqs 9 and 10:
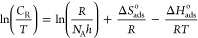
9where *h* and *N*_A_ are the Planck constant (6.626 ×10^–34^ J s) and Avogadro constant (6.022 ×10^23^ mol^–1^), respectively. Figure 6c shows a suitable linear fitting of the experimental data to the transition state, mainly at concentrations below 100 ppm, where the slope, obtained by linear regression, is equivalent to the  value and intersection at . The obtained values of such thermodynamic parameters are listed in Table 5. Positive  values are observed, which indicate the endothermic nature of the adsorption process of C_12–14_TC_1_AmD on the steel surface, i.e., there is a heat adsorption process.^3,9^

**Table 5:** Kinetic Parameters of the Inhibition Process of API 5L X60 Steel in H_2_SO_4_ with and without CI

*C*_INH_ (ppm)	(kJ/mol)	(J/mol K)	*E***_a_** (kJ/mol)
0	42.0	–88.8	44.6
50	18.2	–178.0	20.8
75	22.0	–168.1	24.6

Furthermore, with the addition of 75 ppm of C_12–14_TC_1_AmD, values below −41.8 kJ mol^–1^ are obtained, which indicate the physicochemical absorption of the C_12–14_TC_1_AmD molecules.^64^ It has been reported that, according to the unimolecular equation, a quasi-substitution process between organic and water molecules at the metal interface is possible if the *E*_a_*–* value is approximately equal to the average value of *RT* within the same temperature interval.^65,66^ In the present study, at 75 ppm of C_12–14_TC_1_AmD, the *E*_a_*–* value is 2.6 kJ mol^–1^, whereas the *RT* value is 2.56 kJ mol^–1^, suggesting that at this concentration, the quasi-substitution process could take place. On the other hand, the negative  values indicate that the adsorption of C_12–14_TC_1_AmD provokes the diminution of the system entropy.^63^ In the absence of IL, the highly reactive steel sites generate a surface with a higher chaotic degree, which can be related to the high hydrogen evolution at the metal-medium interface provoked by the increase in the kinetics of the electrochemical reactions; notwithstanding, in the presence of CI, the adsorption of C_12–14_TC_1_AmD on the metal surface reduces the kinetics and promotes higher thermodynamic stability by blocking the active sites. The analysis of the kinetic and thermodynamic parameters confirms that the protective ability of C_12–14_TC_1_AmD against steel corrosion is improved at higher temperatures because the presence of chemical bonds between the CI and metal allows higher stability of the molecular monolayer even with the increasing medium internal energy.

### 3.3 Surface Analysis

**Figure 7:**
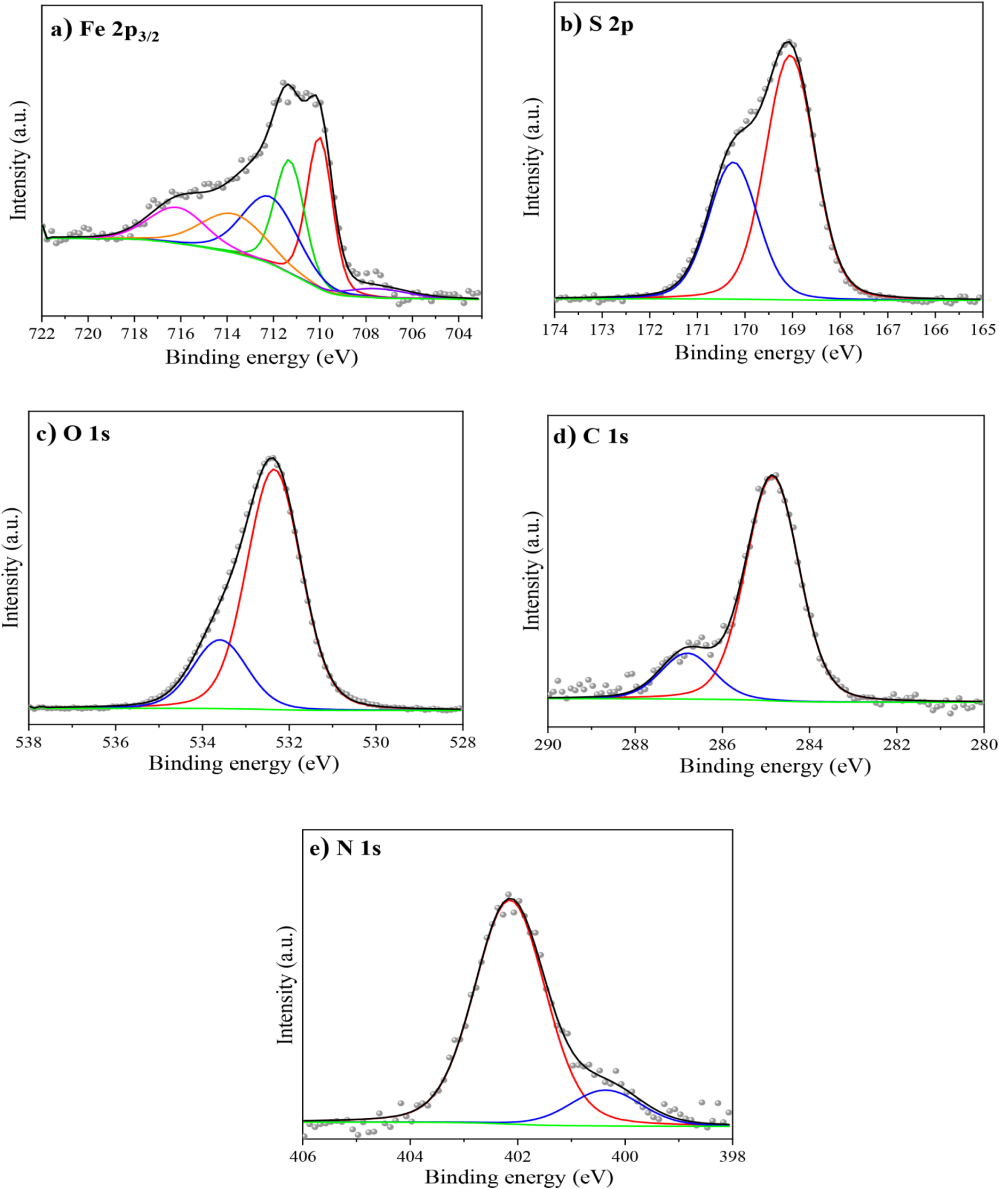
High-resolution XPS spectra of the API 5L X60 steel surface immersed in 1 M H_2_SO_4_ containing 150 ppm of C_12–14_TC_1_AmD: (a) Fe 2p_3/2_, (b) S 2p, (c) O 1s, (d) C 1s, and (e) N 1s.

Figure 7 shows the high-resolution spectra of Fe 2p_3/2_, S 2p, O 1s, C 1s, and N 1s of API 5L X60 steel after being immersed for 4 h in 1 M H_2_SO_4_ at 150 ppm of C_12–14_TC_1_AmD. Table 6 presents the binding energy values, FWHM values, and corresponding assignations of the obtained XPS signals. The Fe 2p_3/2_ spectrum (Figure 7a) shows the Fe^0^ signal at 707.5 eV, related to Fe in elemental state;^67^ in addition, it displays multiplets assigned to Fe^2+^ /Fe^3+^ species with four peaks at positions 710.0, 711.3, 712.2, and 713.7 eV, as well as the corresponding satellite peak. The intense signal at 710.0 eV is associated with “green rust”, whereas the signals at 711.3, 712.2, and 713.7 eV are related to the presence of iron sulfates such as melanterite (FeSO_4_·7H_2_O), rozenite (FeSO_4_·4H_2_O), and szomolnokite (FeSO_4_·H_2_O), which are the characteristic corrosion products in aqueous sulfuric acid in the presence of CIs.^68^

**Table 6:** XPS-Binding Energy of the API 5L X60 Steel Surface in 1 M H_2_SO_4_ Containing 150 ppm of C_12–14_TC_1_AmD

Element	Assignment	Binding energy (eV)	FWHM (eV)
C	C–O/C–N	286.8	1.4
	C–H/C–C	284.8	1.4
Fe	Fe^2+^ 2p_3/2_ Satellite	716.2	2.9
	Fe^2+^ 2p_3/2_(IV) (FeSO_4_·H_2_O)	713.7	3.3
	Fe^2+^ 2p_3/2_(III) (FeSO_4_·4H_2_O)	712.2	2.8
	Fe^2+^ 2p_3/2_(II) (FeSO_4_·7H_2_O)	711.3	1.5
	Fe^2+^ 2p_3/2_(I) [(Fe^2+^Fe^3+^(OH)_12_^–^]^+2^	710.0	1.3
	Fe^0^ 2p_3/2_ (metal)	707.5	3.1
N	–^+^N–R_4_ (N1)	402.1	1.6
	=N–Fe (N2)	400.4	1.6
O	O^2–^ (adsorbed H_2_O)	533.6	1.5
	O^2–^ (FeSO_4_·*x*H_2_O; Fe–O)	532.4	1.5
S	S^6+^ 2p_1/2_ (FeSO_4_·*x*H_2_O)	170.3	1.2
	S^6+^ 2p_3/2_ (FeSO_4_·*x*H_2_O)	169.1	1.2

Furthermore, the peaks at 170.3 and 169.1 eV of the S 2p spectrum (assigned to S^6+^ 2p_1/2_ and S^6+^ 2p_3/2_, respectively) shown in Figure 7b, as well as the peak at 532.4 eV of the O 1s (O^2–^ 1s) spectrum, Figure 7c, confirm the presence of iron(II) sulfates on the steel surface, which are characteristic corrosion products of the corrosive system steel-sulfuric acid^69^ (Table 6).^70,71^

**Figure 8:**
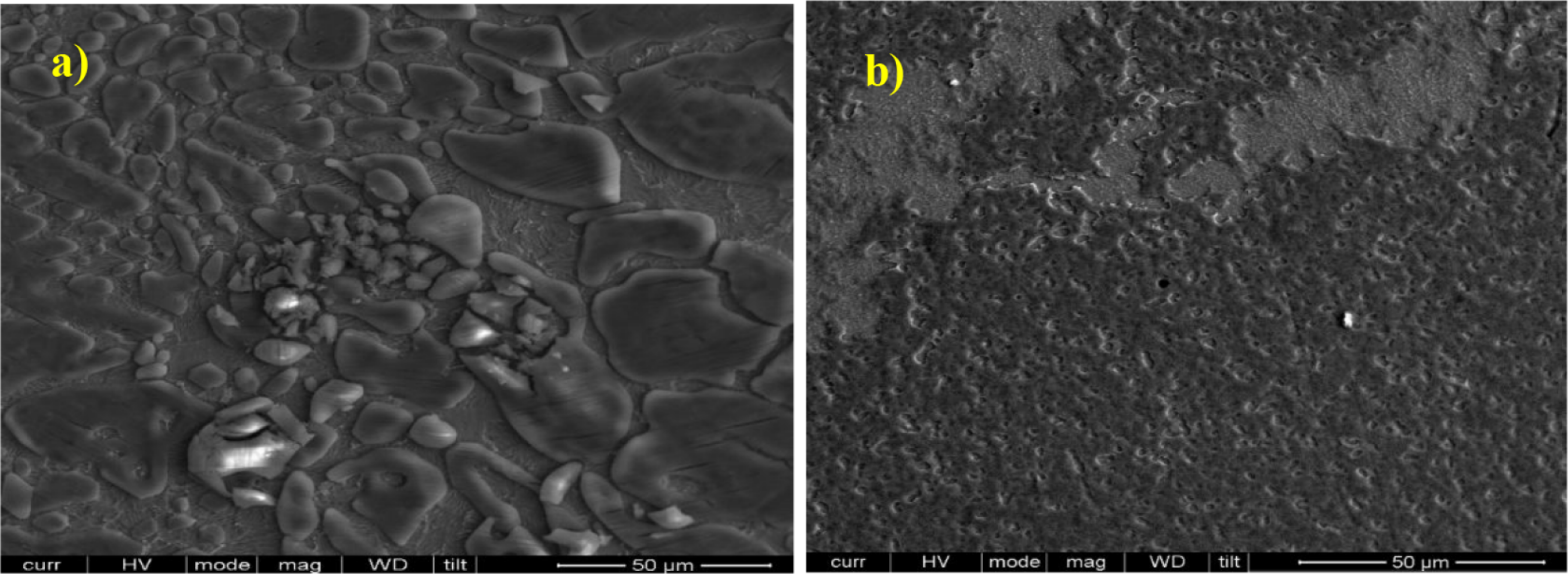
SEM images of API 5L X60 steel after 4 h of immersion in 1 M H_2_SO_4_: (a) in the absence and (b) in the presence of C_12–14_TC_1_AmD at 150 ppm.

A second peak of lower intensity at 533.6 eV for oxygen is attributed either to the carboxylate groups or oxygen present in H_2_O molecules adsorbed on the metal surface.^16,19^ In Figure 7d, the C 1s spectrum displays two peaks: the first one, with a higher intensity at 248.8 eV, was assigned to C–C/C–H bonds, and a second peak, at higher bond energies (286.8 eV), was assigned to C–O/C–N.^16,70,72^ The first peak can be related to carbon present in the long, 14-carbon-atom alkyl chain in the cationic part of C_12–14_TC_1_AmD or to carbon in the anion aromatic rings, whereas the second peak can represent the C–O/C–N bonds in the anionic part (carboxylic group) and in the cationic part (quaternary ammonium) of the CI molecule. Finally, the N 1s spectrum confirms the presence of C_12–14_TC_1_AmD on the metal surface: a peak at 402.1 eV (N1), with higher intensity and related to the quaternary ammonium polar group present in the C_12–14_TC_1_AmD cation, is shown, whereas at higher binding energy (400.4 eV (N2)), a second peak appears. The presence of the latter can be due to different assignations: (i) first, as it has been reported by different authors, this signal comes from N–Fe bonds that indicate the chemisorption of the inhibitor molecule on the metal surface;^6,7,11,67^ (ii) also, it has been ascribed to C=N–C/–NH_2_ bonds.^72,73^ In addition, the high N1/N2 atomic ratio of 5.9 supports the hypothesis that a cation derived from quaternary ammonium interacts with the steel surface mainly through electrostatic interactions or through van der Waals interactions. Figure 8a,b shows the micrographs of API 5L X60 steel after 4 h of immersion in 1 M H_2_SO_4_ at 308 K in the absence and presence of 150 ppm of C_12–14_TC_1_AmD, respectively. The steel sample without CI displays a heterogeneous surface morphology with evident surface damage. The grain boundaries of API steel are evident as well as the presence of particles deposited on these zones, which can be the evidence of two corrosion types: general and intergranular. The presence of corrosion products of the goethite type in the shape of needles is evident in Figure 8a with iron sulfate inclusions. On the other hand, in the micrograph with the presence of C_12–14_TC_1_AmD, Figure 8b, the surface shows a more homogeneous appearance with a lower amount of corrosion products, which are represented by rozenite, melanterite, and “sulfate green rust”. This means that the presence of CI not only diminishes the steel corrosion rate (confirmed by the electrochemical tests) but also changes the path of formation of corrosion products more toward hydrate iron sulfates than toward iron oxyhydroxides.

### 3.4 Theoretical Analysis

**Figure 9:**
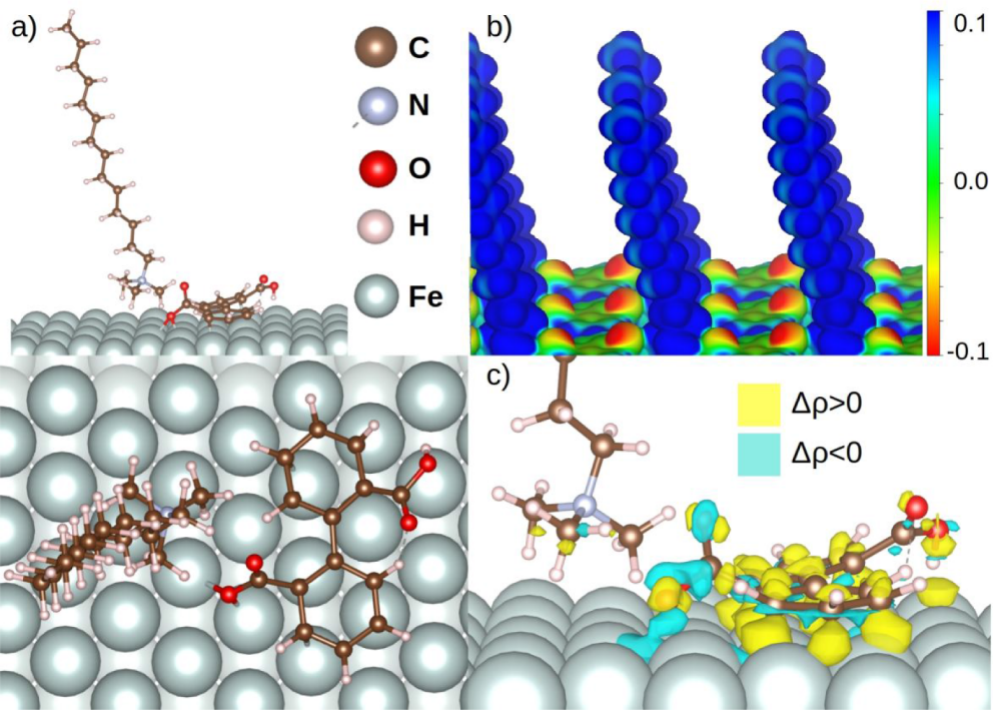
(a) Side (up) and top (down) views of the optimized structure of the C_12–14_TC_1_AmD adsorbed on Fe[110], (b) electrostatic potential mapped on the electronic density surface (isovalue = 0.03), and (c) Δρ isosurface through adsorption (isovalue = 0.025).

The optimized IL structure on Fe [110] shows that the anion is placed close to the surface with Fe–C distances between 2.06 and 2.18 Å, suggesting the formation of covalent bonds, Figure 9a. The cation is stabilized at the position shown in Figure 9a, where the closest hydrogen atoms to the surface show distances above 2.45 Å, which are greater than those reported for the Fe–H covalent bond (1.5–1.8 Å) in various complexes,^74^ suggesting the occurrence of physisorption. The attractive interaction energy between the cation–anion complex and the metal surface is 7.99 eV, mainly due to the covalent bonds between the aromatic rings and the surface. From the electrochemical results, it is known that with the IL adsorption, a highly covering monolayer is formed; the theoretical model represents this state as highly ordered, where after adsorption, the formed C_12–14_TC_1_AmD layer presents a positive electrostatic potential (Figure 9b), which would repel any positive ion from the corrosive medium and avoid its approach to the steel active sites.

**Figure 10:**
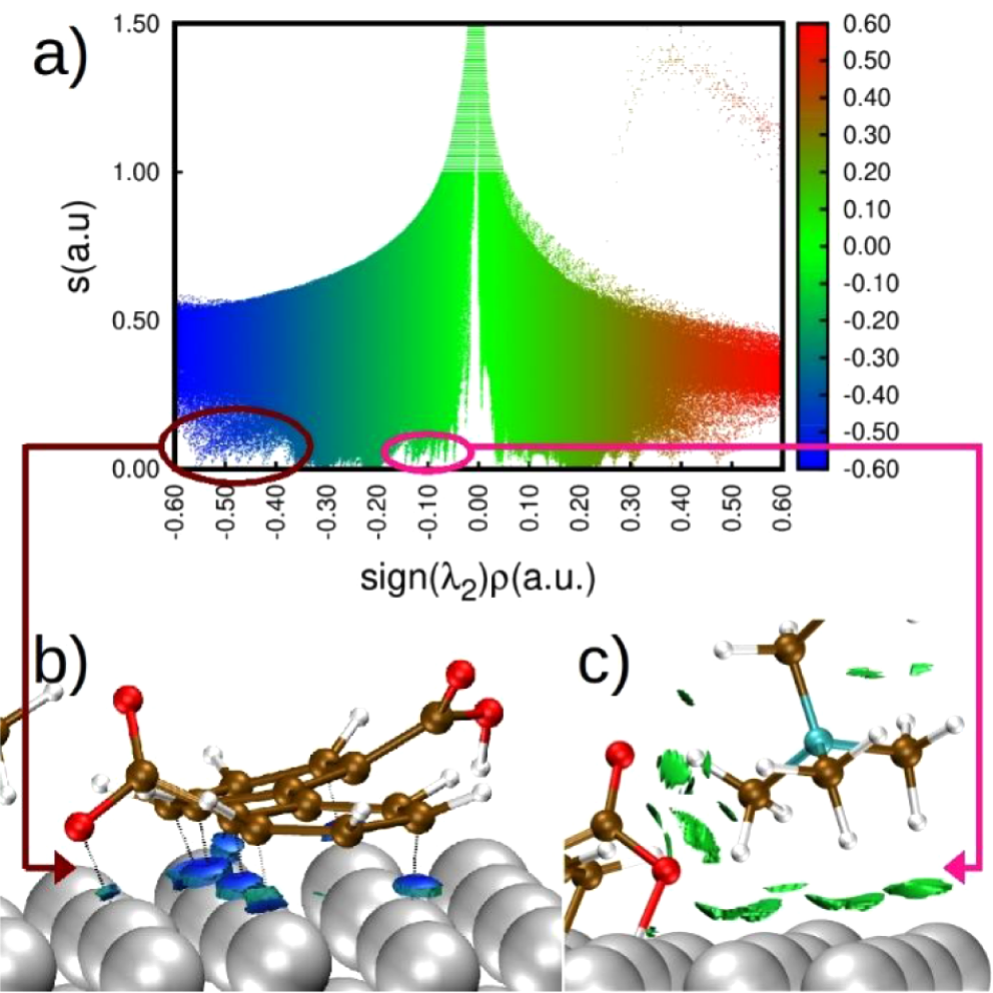
NCI analysis of the IL–Fe surface interactions. (a) RDG vs sign(λ_2_)ρ, RDG isosurfaces of the (b) anion–surface and (c) cation–surface interactions.

To analyze the adsorption mechanism, the calculation of the electronic density change (Δρ) due to adsorption was carried out. Δρ in Figure 9c shows that charge accumulation occurs mainly in the zone of π* orbitals of the rings and d orbitals of the surface Fe atoms, which is the area between Fe–C atoms and where covalent bonds are formed. The charge donation goes from Fe atoms to the aromatic rings and, in turn, from the carboxylate group in the rings and slightly from the σ molecular orbitals through a donation–backdonation mechanism. To localize and classify the interactions between the CI and metallic surface, noncovalent interaction (NCI) analysis was conducted.^75^ The NCI is a topological analysis of density and its gradient, and it stems from the study of noncovalent interactions, but its ability to differentiate strong interactions (covalent, ionic, charge-shift bonds) has been probed. The NCI procedure consists in calculating the reduced density gradient (RDG).^76^ The RDG is zero if an interaction is established, where the smaller the electron density, the weaker the interaction. Binding and nonbinding interactions are differentiated if the sign of the second eigenvalue of the electron density Hessian λ_2_ is negative or positive, respectively. In Figure 10a, all of the binding IL–metal surface interactions are observed like peaks, where RDG is zero. The binding interactions with the aromatic rings are observed in Figure 10b with ρ values of 0.6:0.4 that indicate covalent interactions; even O–Fe interactions are observed. The cation interactions correspond to the green isosurfaces in Figure 10c; by the ρ value (0.05:0.14), it is suggested that these are ionic interactions. Besides, interactions between ions can be observed.

Deeper analysis of the electronic structure of the cation and anion of C_12–14_TC_1_AmD was done to expose the importance of its performance as a CI. The HOMO and LUMO orbitals were identified because these are the energetically preferred sites for donating or accepting charges. Furthermore, the Fukui (*f*+ and *f–*) functions were calculated given that they show the ability to accept or donate electrons.

**Figure 11:**
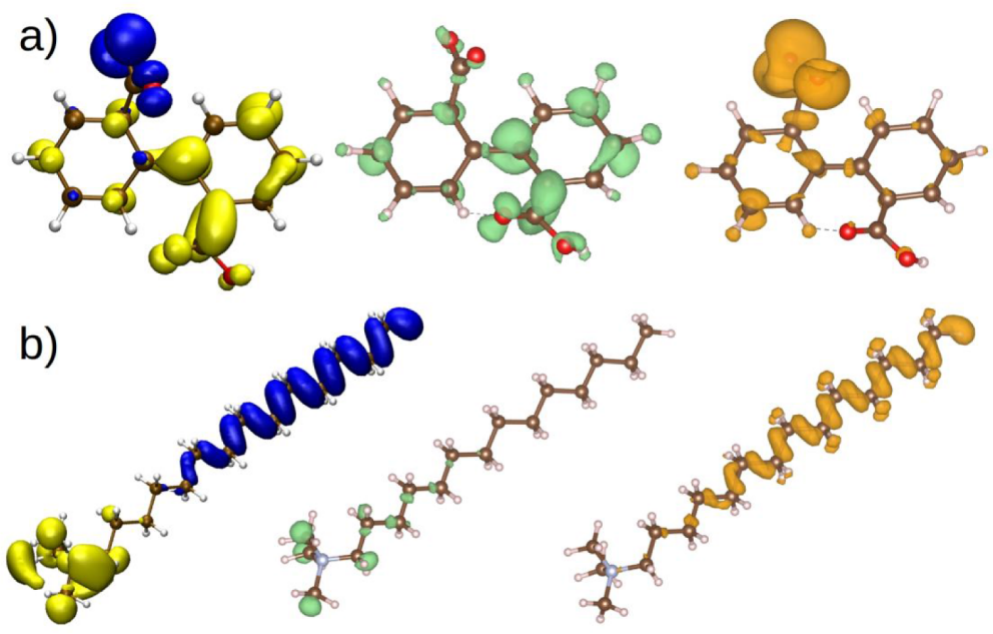
(Left) Frontier orbitals (HOMO – blue, LUMO – yellow), (center) *f*+ function, and (right) *f*– of the (a) anion and (b) cation. Isovalues: 0.06 frontier orbitals; 0.025 anion Fukui functions; and 0.001 cation Fukui functions.

For the anion adsorbed on the metal surface, the donation of electrons takes place by the carboxylic group, where the HOMO is found (Figure 11a, left) and is the region with the highest value of Fukui *f*– (Figure 11a, right). As for the regions that are prone to accept electrons, the LUMO is distributed around both rings (Figure 11a, left) that are capable of having electrons in both of them. The Fukui *f*+ function also displays this behavior (Figure 11a, center). Both the frontier orbitals and Fukui functions support the IL–surface interaction through the aromatic rings and the API X60 steel active sites.

For the cation in the IL C_12–14_TC_1_AmD, the HOMO (Figure 11b, left) is distributed along the aliphatic chain, but *f–* (Figure 11b, right) shows almost negligible maximal values (∼0.001), which indicates that it does not have the capacity to donate electrons. The removal of more electrons requires a high amount of energy. Finally, the LUMO is distributed in the amino group (Figure 11b on the left), including N, which is related to the capacity to accept electrons from the anion, precisely in the amino group, as shown by *f*+ (Figure 11b, center).

### 3.5 Inhibition Mechanism

**Figure 12:**
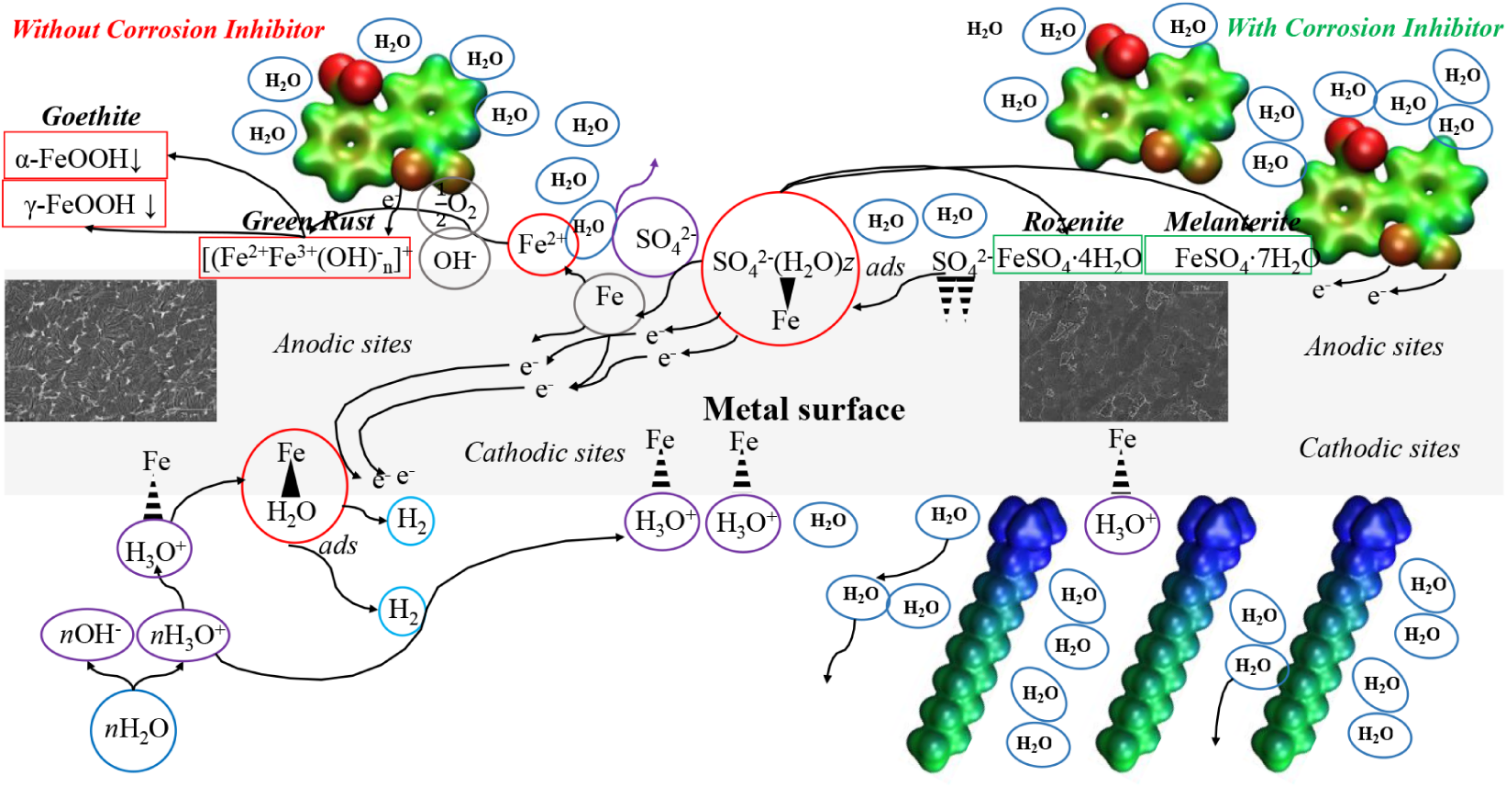
Corrosion inhibition mechanism carried out by C_12–14_TC_1_AmD.

Based on the results obtained in the experiments and theoretical calculations, it is possible to propose a corrosion mechanism, as shown in Figure 12. The CI molecules, which are dissociated in the aqueous sulfuric acid dissolution, approach the surface-active sites working as a mixed-type inhibitor on the cathodic and anodic sites on the metal surface.

In the absence of CI, the iron cations (Fe^2+^) detach from the metal surface because the iron sulfates dissolve in the aqueous medium, provoking further formation of oxyhydroxides (α-FeOOH, γ-FeOOH, and γ-Fe_2_O_3_), mainly goethite (α-FeOOH) (eqs 10 and11):
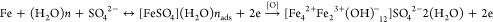
10
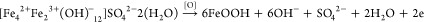
11

The theoretical calculation confirms that when the anion, represented by diphenic acid, approaches the iron surface and both rings are placed on the same plane, LUMO gets distributed around both aromatic rings, which are capable of accepting electrons, whereas the donation of electrons occurs by the carboxylic group, where HOMO is located. The molecular anionic part changes its orientation, according to theoretical analysis, in such a way that both aromatic rings become oriented parallel to the metal surface, thus facilitating the backdonation of electrons on the metal anodic sites and forming chemical bonds between the CI anionic part and the metal surface.

The values corresponding to corrosion inhibition and activation and free Gibbs energies increase with temperature up to 39.2 kJ mol^–1^, thus confirming that the adsorption process of CI molecules occurs through a chemisorption trend, forming a dielectric layer according to the EIS results. In addition to the XPS and SEM results, where not only the CI presence through the C and N signals, but also the occurrence of “sulfate green rust” and hydrate iron sulfates such as melanterite and rozenite, without the presence of goethite, were detected, it is proposed that CI molecules on the anodic sites slow down the dissolution and desorption of corrosion products such as iron sulfates and “green rust” on/from the metal surface to the aqueous acid medium (eq 12).
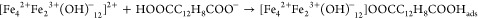
12

On the cathodic sites, the CI cationic part approaches the surface, displacing the water molecules and competing with the hydronium cation for the active sites, occupying the geometric space that is much bigger than itself, thus slowing down the hydrogen evolution reaction chain on the metal surface (eq 13).
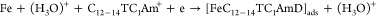
13

This CI action mechanism confirms that C_12–14_TC_1_AmD is a mixed-type inhibitor.

## 4 Conclusions

The IL trimethyldodecyl(tetradecyl)ammonium diphenyl-2,2’-dicarboxylate behaved as a CI of API 5L X60 steel in 1 M H_2_SO_4_. The maximal inhibition performances were achieved at 150 ppm with IEs of 84, 94, and 96% at 298, 308, and 318 K, respectively, according to the PDP and EIS electrochemical techniques.

The adsorption mechanism of the CI on the steel surface allowed the formation of a monolayer, where two processes were involved: (a) cation-based physisorption and (b) anion-based chemisorption. In addition, the monolayer was stable during the temperature increase, according to the free Gibbs energy from 35.7 to 39.1 kJ mol^–1^.

The CI adsorption was confirmed by surface analysis (XPS and SEM), showing that in the presence of CI, corrosion products such as iron sulfates (melanterite and rozenite) were not detached from the metal surface, protecting it, whereas in the absence of CI, the main corrosion products were goethite-type iron oxyhydroxides.

Finally, the theoretical calculations of C_12–14_TC_1_AmD confirmed that the chemical configuration of the cation and anion in the structure of this IL evaluated as the CI allowed its adsorption through a mixed-type mechanism on the surface of the API 5L X60 steel; for this reason, its IE was boosted by the increasing temperature.
